# Emerging Feature Extraction Techniques for Machine Learning-Based Classification of Carotid Artery Ultrasound Images

**DOI:** 10.1155/2022/1847981

**Published:** 2022-05-12

**Authors:** S. Latha, P. Muthu, Samiappan Dhanalakshmi, R. Kumar, Khin Wee Lai, Xiang Wu

**Affiliations:** ^1^Department of Electronics and Communication Engineering, SRM Institute of Science and Technology, Kattankulathur, Chennai 603203, India; ^2^Department of Biomedical Engineering, SRM Institute of Science and Technology, Kattankulathur, Chennai 603202, India; ^3^Department of Biomedical Engineering, Faculty of Engineering, University Malaya, Kuala Lumpur 50603, Malaysia; ^4^School of Medical Information Engineering, Xuzhou Medical University, Xuzhou 221000, China

## Abstract

Plaque deposits in the carotid artery are the major cause of stroke and atherosclerosis. Ultrasound imaging is used as an early indicator of disease progression. Classification of the images to identify plaque presence and intima-media thickness (IMT) by machine learning algorithms requires features extracted from the images. A total of 361 images were used for feature extraction, which will assist in further classification of the carotid artery. This study presents the extraction of 65 features, which constitute of shape, texture, histogram, correlogram, and morphology features. Principal component analysis (PCA)-based feature selection is performed, and the 22 most significant features, which will improve the classification accuracy, are selected. Naive Bayes algorithm and dynamic learning vector quantization (DLVQ)-based machine learning classifications are performed with the extracted and selected features, and analysis is performed.

## 1. Introduction

Cardiovascular illnesses are the chief reason for death as per the World Health Organization. Accurate measurement and analysis of the carotid artery provide early identification of stroke and atherosclerosis diseases, which are caused by plaque deposit. In clinical diagnosis, plaque morphology and stenosis severity characterization may be diagnosed using noninvasive ultrasound imaging [1]. Experienced radiologists outline the layer thicknesses and plaque deposition buy may vary depending on the person. Automatic segmentation and classification of the carotid artery ultrasound images are necessary for accurate and early assessment of the disease.

Biochemical risk factors and vital signs include high C-reactive protein, glycohemoglobin, low-density lipoprotein cholesterol, serum creatinine, factor alpha, platelet-to-lymphocyte ratio, and neutrophil-to-lymphocyte ratio. Biomechanical risk factors include increased plaque area, low stenosis grade, increase in IMT, presence of in-plane curvature, abnormal shear stress, C-shaped ICA, low wall shear stress, and reversal flow. The related diseases because of plaque formation in the carotid artery are diabetes, hypertension, plaque hemorrhage, head and neck radiotherapy, chemotherapy, coronary artery diseases, peripheral artery diseases, and low ankle-brachial index [2, 3].

Feature extraction reduces the data dimensionality by removing the redundant features, hence improving the training and inference speed. Nonredundant significant features from the ultrasound images of the carotid artery are extracted and used by machine learning (ML) algorithms to classify the image as abnormal or normal. Using additional features might cause overfitting, and less count features may make the data underfit. A good machine learning model may be built with relevant extracted and selected features from the image. Blood flow features like end-diastolic height, resistivity index (RI), maximum systolic height, pulsatility index (PI), spectral broadening index, and height width index help in plaque identification. Texture, grayscale median (GSM), entropy, coarseness, Hurst coefficient, etc. are some of the useful features [4, 5]. The feature mapping to the classes in a computer-assisted diagnostic (CAD) system is called classification.

Loizou et al. proposed a texture feature variation in carotid artery ultrasound video to recognize the biomarkers of the plaque [6]. Followed by denoising and segmentation, the systole and diastole features were extracted from the video sequence. For the normal, symptomatic, and asymptomatic groups, a higher grayscale median was observed in systole and diastole cardiac cycles. Plaque texture features and grayscale median (GSM) were considerably diverse for the two classes. A multiresolution texture classification technique was proposed by Nikolaos et al. to classify the B-mode carotid artery ultrasound image as with or without plaque deposit [7]. Wavelet packet, stationary wavelet, discrete wavelet, Gabor transform, and other basis function decomposition schemes showed effectiveness in identifying the disease. Appropriate features were selected using deviation in values of the features and using a nonlinear correlation coefficient for thresholding. He proved that biomechanical forces affected texture analysis, and they showed horizontal directionality.

Prostate cancer inventions in transrectal ultrasound images by an automatic segmentation approach were proposed by Wang et al. [8]. The low layers of the convolutional neural network (CNN) are suppressed, and the prostate features in the deep layers are increased to identify, select, and influence the multilevel features from the various layers so that each layer features are enhanced. Layer-wise attention is used for enhancing the features in individual layers. The steps are generating attentive maps in layers, concatenate single-layer feature map to multilayer feature maps, and kernel arrangement in the three convolutional layers. The kernels used in the first two convolutional layers are 3 × 3 × 3, and the last kernel has 1 × 1 × 1 layer. From the learned samples in the folds, hyperparameters were identified by cross validation.

A feature-guided denoising CNN was proposed by Dong et al. [9] for removing noise and improving the image quality of portable ultrasound images. A feature masking layer was proposed for hierarchical noise removal in layers. Feature extraction was performed by an explainable artificial intelligence procedure. The highest important features were observed, and the other less important features were also partially preserved by guided backpropagation to map the neural network's choice. The feature images were combined with the Laplacian pyramid fusion method. BI-RADS feature extraction for characterizing the breast cancer lesion, which is continuously varying during the ultrasound sequence, was proposed by Sellami et al. [10]. The analysis was performed for the varying images with morphological and texture features. They concluded that the feature extraction should be performed in many slices of similar mass to get the optimal segmentation results. Slice selection depends on the radiologist, and another slice may have the necessary useful information, which should not be missed for diagnosis.

Siavash et al. proposed a method to quantify morphological features, which helped in classifying tumor cells and disease identification [11]. By conserving the bigger vessel trunks, morphological parameters like tortuosity were measured. They developed an approach to augment the tumor microvessels with ultrafast ultrasound imaging. Wang et al. used a pretrained deep convolutional neural network (DCNN) and developed a multinetwork feature extraction model, which also performed dimensionality reduction and trained an ensemble support-vector machine [12]. Followed by preprocessing of the histological data by scale variation and color improvement, features in many levels were extracted by four trained DCNN. Using dual-network orthogonal low-rank learning, feature selection is performed for overfitting modifications and performance boosting of the method.

Zhou et al. proposed an involuntary measurement with phase images for spine curvature using three-dimensional ultrasound volume projection images [13]. Bony feature extraction by twofold thresholding strategy with asymmetric and symmetric measures information using phase congruency was performed. When the signal Fourier components are hugely in-phase, they can extract Mach bands, roof, line edges, and step edges.

Li and Liu proposed a genetic algorithm using information flow for the Naive Bayes classifier. From the view of correlation and angle of causality, a new weight measure criterion is produced by information flow. The approach provided improved classification accuracy compared with other attribute-weighting approaches with Bayes classifiers. The first, third quartile, and median values were higher for the proposed classifier [14]. Zhang et al. proposed a LVQ with a modular reconfigurable pipelining architecture for object recognition and image compression [15]. The method was verified in a 65 nm CMOS prototype chip. Reduced computation time and integration density efficiency were achieved by the approach.

Feature extraction and selection of the carotid artery ultrasound images are performed to assist ML-based classification. The best significant features are selected by a divergence approach and principal component analysis (PCA). The images are classified as with or without plaque deposit, for early identification of the presence of stroke. This study's layout is as follows.  Section 2 gives the methodology of feature extraction, selection, and classification, Section 3 explains the results and discussions, and Section 4 concludes the study.

## 2. Methodology

This segment describes the feature extraction of the carotid artery ultrasound images. Appropriate features are selected after extracting the image features. The selected features are classified using Naive Bayes and DLVQ machine learning algorithms, and the classification performances are analyzed.

### 2.1. Carotid Artery Ultrasound Images

Ultrasound imaging is economically affordable, noninvasive, and continuously improving in image quality that will provide early diagnosis of arterial disease. Intima-media layer thickness, lumen diameter, and texture characterization are necessary for the assessment of cardiovascular illnesses. The ultrasound images are affected by speckle noise and should be effectively denoised, so that image quality is preserved and the useful features are not lost. Curvelet decomposition-based denoising is performed for noise removal.

**Figure 1:**
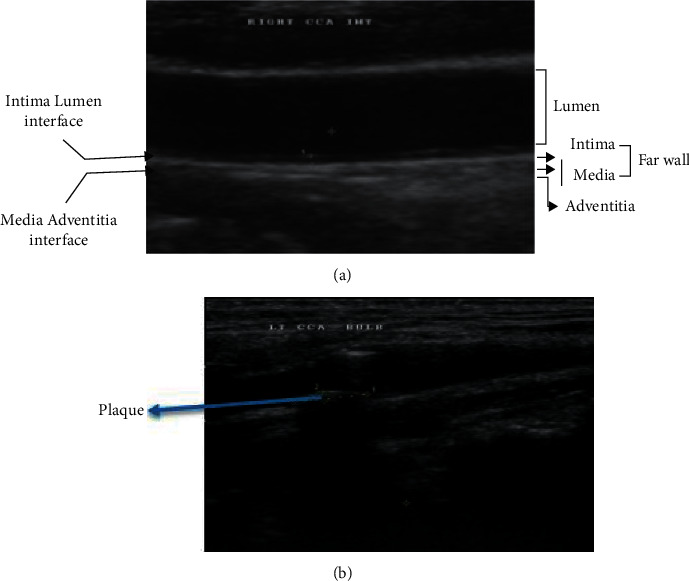
Sample carotid artery ultrasound image (a) with plaque and (b) without plaque.

The carotid artery is an artery in the neck region, which transports pure blood from the heart to the brain and the front of the face. It contains the common carotid artery (CCA), which splits into external carotid artery (ECA) and internal carotid artery (ICA).  Figure 1(a) presents the carotid artery structure, where ICA provides blood to face and ECA to the brain. The bifurcation is a more plaque-prone area.

Figure 1 presents the sample carotid artery ultrasound image with and without plaque deposit. The deepest smooth layer is intima, muscular middle layer media, and the outer layer is adventitia. Lumen is the inner core of the artery. Atherosclerosis is the narrowing of the artery lumen because of fat and calcium deposit in the artery wall, which may lead to thinning of blood flow as indicated. The local thickening in the artery because of fat deposition is called plaque formation. The intima-media thickness (IMT), lumen-intima (LI) interface thickness, and media-adventitia (MA) interface thickness influence the carotid plaque thickening. IMT measurements for a healthy person are from 0.2 to 0.25 mm.

Carotid artery disease identification includes plaque characterization and layer thickness measurements on age, gender, environmental conditions, and other habits of the person. The increased plaque thickness in the carotid is considered as an early indicator of future cardiovascular risks like stroke and atherosclerosis. The artery is at distance of 1 to 3 cm from the skin and so can be analyzed with imaging techniques.

### 2.2. Database Creation

Ethical clearance is obtained from SRM Medical College Hospital and Research Centre, Kattankulathur, Tamil Nadu, India, to get carotid artery ultrasound images. Carotid artery ultrasound B-mode image datasets are collected from SRM Medical College Hospital and Research Centre, Kattankulathur, Chennai, and Bharat Scans, Chennai.

Three hundred and sixty-one carotid artery ultrasound images were obtained, out of which 202 are normal images without plaque deposit and 159 images are with plaque deposit in the artery layers. About 75% (270) of data are used for training and 25% (91) for testing the machine learning algorithms.

### 2.3. Feature Extraction

**Figure 2:**
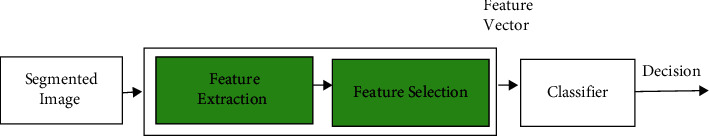
Block diagram of classification system.

Feature extraction and selection prevent overfitting of the training data and poor generalization of the new samples as given in Figure 2. Effective model construction for machine learning depends on the suitable optimized features. For a given feature set *F*={*f*_1_, *f*_2_,…, *f*_*n*_}, the features to be selected should maximize the learner algorithms' ability to classify the carotid image as symptomatic or asymptomatic. *F* should be a function maximizing the scoring function. The features are normalized or translated in the range [0 1]. Thus, the data are aligned with similar distributions and help in model fit. *Z* score normalization procedure is used, which is a standard score technique. Population mean is deducted from the individual data and uniformly divided by the population standard deviation.

Despite variations in class, a useful feature should remain unchanged. Feature extraction categories are based on nontransformed and transformed structural characteristics, and structural and graph descriptors. For high-dimensional feature space, computational complexity increases [16]. A kernel function is made to record the nonlinear data into a higher dimensional space. Kernel is a dot product in high-dimensional space. Thus, all computations are made in the low space dimension. The kernel function is given by

(1) $$\begin{array}{c} K\left(x,x^{\prime }\right)=\;\vartheta {\left(x\right)}^{T}\vartheta {\left(x\right)}^{\prime }, \end{array}$$

*K* is the inner product of the vectors *x* and *x*′ in the low-dimensional feature space for a function  *ϑ*. At an instant *y*, with slope *w*_0_, the input's hyperplane is given by

(2) $$\begin{array}{c} y\left(x\right)=\text{sign}\left[\sum_{i=1}^{M}y_{i}K\left(x,x^{\prime }\right)+w_{0}\right]. \end{array}$$

Feature selection is to identify the relevant properties of the image for data reduction and reduction in feature set, and to improve accuracy for performance improvement and proper understanding of the data for image analysis. Features may be subdivided based on their relevance.

The extracted features may belong to any of the following categories, which assist in deciding whether it is a significant feature.

(i) A feature *X*_*i*_ is irrelevant for all the subsets belonging to *S*_*i*_.
  (3) $$\begin{array}{c} P\left(X_{i},Y|S^{-i}\right)=P\left(X_{i}|S^{-i}\right)P\left(Y|S^{-i}\right). \end{array}$$
These are the features with very less or zero probability calculated by Kullback-Leibler divergence.
(ii) A feature *X*_*i*_ is nearly unrelated with approximation value *μ* > 0, for subsets *S*_*i*_
  (4) $$\begin{array}{c} \text{EMI}\left(X_{i},Y\right)\leq \mu . \end{array}$$
For *μ* = 0, the feature is surely irrelevant.
(iii) A feature *X*_*i*_ is separately inappropriate if the significance threshold *φ* ≥ 0.
  (5) $$\begin{array}{c} \text{MI}\left(X_{i},Y\right)\leq \phi . \end{array}$$
(iv) A feature *i* is possibly nearly inappropriate based on *D* index computed with *n* number of examples, with approximation *ϕ* ≥ 0 and risk *δ* ≥ 0.
  (6) $$\begin{array}{c} P\left(C\left(i,m\right)>\phi \left(\delta ,m\right)\right)\leq \delta . \end{array}$$
(v) A subgroup *V* of features is certainly adequate if it also belongs to the complementary of the subset.
  (7) $$\begin{array}{c} \begin{array}{c} i\\ v \end{array}P\left(Y|V\right)=P\left(Y|X\right). \end{array}$$
(vi) A subgroup *V* of features is roughly adequate with approximation level *ϕ* ≥ 0 when
  (8) $$\begin{array}{c} \text{DMI}\left(V\right)\leq \phi . \end{array}$$
If *ϕ* = 0, the subset is surely sufficient.
(vii) A subset *V* of features is minimally approximately sufficient with approximation level *ϕ* ≥ 0, and other smaller subsets of *ϕ* does not exist.

From the carotid artery dataset, more artery characteristic-related features along with the standard features are extracted to analyze the image and identify whether the person has plaque deposit. The dataset was enhanced and labeled by radiologists for accuracy [17], and 63 shape, texture, morphology, histogram, and correlogram-based features are extracted and analyzed with the machine learning techniques.

**Figure 3:**
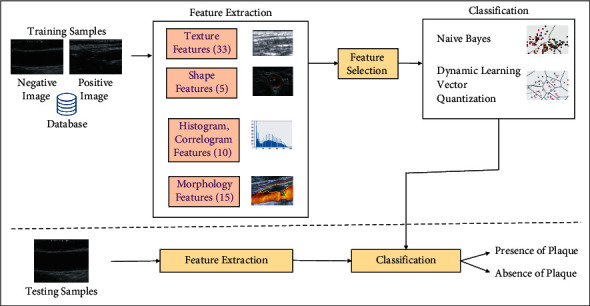
Feature extraction, selection, and classification of carotid artery ultrasound images.

Along with the existing features, plaque morphology-based features extracted are plaque power spectra for all the three intensity variations (low, medium, and high), namely, shape, connectivity, convexity, plaque size, lipid core, presence of intraplaque hemorrhage, smooth lumen surface indicating no risk, and rough lumen, which may lead to stroke and plaque volume.  Figure 3 gives the procedure followed for feature extraction, selection, and classification of the carotid artery ultrasound images.

### 2.4. Extracted Features

Features are extracted from the carotid artery images in the database following preprocessing followed by segmentation steps. The image is noise-free and contrast enhanced, assisting in the quantization of the image features. The below sixty-three features are extracted from the carotid artery ultrasound image database.

Table 1 gives the list of the extracted features. Thirty-three texture features, five shape features, ten histogram- and correlogram-based features are extracted. Fifteen plaque morphology-based features were extracted from the carotid artery ultrasound image database. A detailed explanation of the extracted image features is given below.

#### 2.4.1. Texture Features

Texture in the image is computed from the gray-level co-occurrence matrix (GLCM), which uses spatial difference among the pixels, which are in pairs with a certain distance, relation, and direction [18, 19]. Inertia, energy, correlation, entropy, and homogeneity information are extracted from the matrix. It is proved that the directions 0°, 45°, 90°, and 135° are less dominant for GLCM in medical images, requiring less computation at these angles. For image *I*(*k*, *k*), the co-occurrence matrix is given by

(9) $$\begin{array}{c} C_{M}=\sum_{i=1}^{k}\sum_{j=1}^{k}\left\{\begin{array}{c} 1,\text{ if }I\left(i,j\right)=k,I\left(j+D_{x},j+D_{y}\right)=k,\\ 0,\text{ otherwise}, \end{array}\right. \end{array}$$

where *D*_*x*_=*D*.  cos(*θ*), and *D*_*y*_=*D*.  sin(*θ*), *θ* being the offset defining matrix direction from the central pixel, and *D*, the distance from the central pixel. For the given angle *θ*, the parameters computed are as follows:

(10) $$\begin{array}{c} \text{contrast}=\sum_{a=1}^{L}\sum_{b=1}^{L}{\left(a-b\right)}^{2}C_{M},\\ \text{correlation}=\sum_{a=1}^{L}\sum_{b=1}^{L}C_{M}\left[\frac{\left(a-\mu_{a}\right)\left(b-\mu_{b}\right)}{\sqrt{\left(\sigma_{a}^{2}\sigma_{b}^{2}\right)}}\right],\;\\ \text{energy}=\sum_{a=1}^{L}\sum_{b=1}^{L}C_{M}^{2}\;,\\ \text{homogenity}=\sum_{a=1}^{L}\sum_{b=1}^{L}\frac{C_{M}}{1+\left|a-b\right|}\;. \end{array}$$

Gabor wavelet texture features are computed in precise directions and frequencies. Statistical features like mean, median, kurtosis, and skewness were computed. LBP gives the characteristics of the texture's spatial structure. GLCM analyzes the pixel pair's gray-level distribution and is known as a second-order histogram. Gray-level difference statistics (GLDS) extracts the texture features mean, entropy, angular second moment, contrast, and homogeneity representing the difference between the pairs of average gray levels. For *d* = 1, the mean displacement values were computed.

The following are the relation between texture and statistical characteristics.

- For dis-similar pixel pair in coarse texture region, contrast is high, representing considerable variation in gray level.
- A high-energy feature value represents the textural uniformity of the image.
- Uniform GLCM is represented by a large entropy value, which represents nonhomogeneity or degree of disorder.
- Homogeneity of the pixel pair is small for different gray-level pixel pairs.
- Pixel's neighborhood influence represents the correlation.

The pixel statistical properties given by the statistical feature matrix (SFM) are coarseness, roughness, periodicity, and contrast. Pixel's self-similarity is represented by fractals in Euclidean space for a bounded set *A* for *N* overlapping copies of itself. For a scaling ratio *s*, the fractal dimension is given by

(11) $$\begin{array}{c} D=\frac{\log\;N\left(s\right)}{\log\left(1/s\right)}. \end{array}$$

A rough texture has less fractal dimension texture analysis (FDTA) at image resolution 4. Radial and angular sum of discrete Fourier transform for Fourier power spectrum textural feature is computed [20]. The texture's visual properties are projected in the neighborhood gray-tone difference matrix (NGTDM), given by strength, complexity, coarseness, and contrast. The absolute gradient value is more when the pixel intensity moves from black to white, and it is less when it moves from dark gray to a lighter shade. Dark to light shade movement is indicated by a positive gradient, and light to dark shade is represented by a negative gradient [21]. Mean and variance are computed from the absolute gradient texture feature. It measures the mean gray-level changes through the image and how far the pixels are from the mean deviation. Pixels of the same gray level in a certain route are given by the run-length matrix.

#### 2.4.2. Shape Features

Shape-based similarity among the pixels is extracted by shape features. It is a content description based on and performed by feature extraction and likeness measurement among the extracted features. Region-centered shape feature projects the contour characteristics in the entire image region, and contour-based shape feature gives the shape in the contour alone. Contour-based shape features extracted are sharpness, complexity, length irregularity, aspect ratio, and circularity. Contour detection from the edges is possible with these rotation, translation, and scale-invariant shape features [22–24].

(12) $$\begin{array}{c} \text{sharpness}=\sum \frac{\text{max}\left(0,1\right.-\left({\left(2\left|\theta -\pi \right|/\pi \right)}^{2}\right)}{n},\\ \text{complexity}=\frac{10^{-3}}{n},\\ \text{length irregularity}=\frac{\sum \left|L_{i}-L_{i+1}\right|}{K}. \end{array}$$

For *n* > 3, *K* = 2*P* and for *n* = 3, *K* = *P*, which is the length of the polygon segment and the next segment.

(13) $$\begin{array}{c} \text{aspect ratio}=\frac{p_{1}+p_{2}}{C},\\ \text{circularity}=\frac{4\;pA}{P^{2}}, \end{array}$$

where the segment boundary enclosed polygon sides *n*, discontinuity angle *θ*, the perimeter of the polygon bounded by segment border *P*, and the most significant perpendicular distances to the boundary *p*_1_ and *p*_2_. *X* and *Y* coordinates of the picture frame, area of ROI, perimeter, and perimeter^2^/area of ROI were computed to find whether the complexity and size of the carotid image shape help in identifying plaque deposit.

#### 2.4.3. Histogram and Correlogram Features

For the continuous pixel data, the histogram is its frequency distribution representation. Gray-level histogram of segmented ROI for the carotid image for thirty-two same width bins was computed. The plaque histogram feature effectively represents the plaque characterization. The multiregion histogram is computed for equidistant ROI to check whether the plaque outer region signifies disease progression. Grayscale median (GSM) derived from the histograms first-order statistics and entropy represents the echogenicity and arterial wall ROI's randomness. For the image array *Y*, *T*_2_ histogram is computed for a known mean scalar *A*. Image data are obtained from the first-order histogram with *n*^th^ feature *y*_*n*_(*i*, *j*) in the local region *M*. Let us consider *y*_*n*_ constitute discrete values only. The distance vector, which is the gray-level difference between the two pixels, is given by

(14) $$\begin{array}{c} d=\left(\Delta_{x},\Delta_{y}\right), \end{array}$$

for integers Δ_*x*_ and Δ_*y*_. At distance *d*, the gray-level difference is given by

(15) $$\begin{array}{c} Y\left(d\right)=\left|I\left(i,j\right)-I\left(i+\Delta_{x},j+\Delta_{y}\right)\right|. \end{array}$$

The HOG feature derived from DCT gives the details about corners and edges in the image. Gradient and orientation information of the artery layer localized region is extracted after separating the image into smaller regions. The histogram of each region is separately computed from the gradients and orientations of pixel values. For directions *G*_*x*_ and *G*_*y*_, the gradient magnitude is given by

(16) $$\begin{array}{c} \text{gradient magnitude}=\sqrt{G_{x}^{2}+G_{y}^{2}}. \end{array}$$

The orientation of each pixel is given by

(17) $$\begin{array}{c} \tan\;\phi =\frac{G_{y}}{G_{x}}. \end{array}$$

The number of HOG features is dynamic based on the size of each region. Based on the gradient and direction, the weighted HOG is generated. To prevent aliasing problems, in orientation and position centers, bilinear vote interpolation is performed.

Statistics and spatial distribution of the features are represented by a type of histogram called correlogram [25]. Pixel gray intensity from the center is computed to get the correlogram of the image. The distance of each pixel from the center is identified from which equidistant pixels histograms were computed. The calculated distance to the maximum distance difference is divided by the normalization factor, which gives the correlogram comparison. The texture and shape features are normalized, whereas histogram and correlogram features are used without normalization.

#### 2.4.4. Morphology Features

The presence of structural elements at different scales is identified by morphological features. The near-isotropic detection structural elements are represented by + in the structural pattern of the plaque. At the two morphological sets, mean cumulative distribution functions (CDFs) and the mean probability density functions (PDFs) are computed. Plaque component-based multilevel decomposition describes the morphology of the image [26, 27]. The normalized image is thresholded at low, medium, and high intensities. The unstable plaque is indicated by low dark components, and the bright region indicates stable plaque. The plaque power spectra for all the three intensity variations are used as a feature for plaque classification. The hypergeometric function used to capture the morphology of the image by the *n*_th_-order Krawtchouk classical polynomial *k* is given by

(18) $$\begin{array}{c} k_{m}\left(x;p,N\right)=\sum_{k=0}^{N}a_{k,m,p}x^{k}={\text{aF}}_{1}\left(-m,-x;-N;\frac{1}{P}\right). \end{array}$$

For a given *x*, with *m* = 0 to *N*, *N* > 0, *p* ∈ (0,1) if the hyperbolic function *F*_1_. This represents the geometrical structure of the artery and the carotid plaque with details of shape, connectivity, and convexity. Clinical diagnosis depends on the artery wall morphology as a weighted map of the weighted original image. If the segmentation results are poor, the contour morphology may miss some details, leading to improper diagnosis. The morphological stream of feature extraction selects all the morphological features required for learning and classification like plaque size, lipid core, and presence of intraplaque hemorrhage [28]. Plaque destabilization leads to plaque rupture, and the disrupted plaque constitutes a thrombus in the distal artery layers. The volume of plaque in the carotid artery is not a clear indicator of the degree of stenosis. Ischemic symptoms can be identified by the plaque morphological features. In spite of any stenosis degree, the features may cause stroke risk. Thus, the morphological features and the degree of stenosis together give the plaque vulnerability characteristics. A smooth lumen surface indicates no risk, and a rough lumen is considered to lead to stroke.

Blood pressure and plaque rupture cause intraplaque hemorrhage, and it is closely related to cerebrovascular events. The susceptible plaque has a thin fibrous cap, which covers a lipid-rich necrotic core containing inflammatory cells. The artery attempts to maintain a uniform plaque diameter concerning the lumen. Positive remodeling tends to increase the diameter, and adverse remodeling tends to decrease the diameter, which is called stenosis [29, 30]. Plaque volume is proved to be a more significant feature to identify the disease progression and vulnerability than the degree of stenosis.

Echolucent plaque, which contains big white blobs, is considered to be dangerous. Calcified and collagen-stuffed plaque is stable and has very less chance of rupture with a tiny high-intensity image. A lipid with scattered components in the low-intensity image can be characterized as asymptomatic. Thus, unstable plaque is an effect of the thin fibrous cap. From the morphological features extracted, it is understood that plaque instability may not lead to stenosis but reduces blood flow in the artery.

### 2.5. Feature Selection

A robust subset of the extracted features is selected to reduce computational complexity and improve classification accuracy. Feature selection aims at dimensionality reduction, retaining the discriminatory information from the images. The maximum discriminant features from the extracted features are carefully chosen depending on the subsequent method.

The distance among two classes for all the features are generated as below for mean *m*_1_, *m*_2_ and standard deviation *σ*_1_, *σ*_2_.

(19) $$\begin{array}{c} \text{distance}=\frac{\left|m_{1}-m_{2}\right|}{\sqrt{\sigma_{1}^{2}+\sigma_{2}^{2}}}. \end{array}$$

Significant features are identified using the distance measure, and increased distance means more the significance. Sixty-five features are extracted, from which twenty-two most relevant features are considered for the classification process. Along with this method, the principal component analysis (PCA) method for the selection of features is performed. Eigenvalues are used for deriving the principal components. Uncorrelated feature set and principal components are achieved from the correlated ones, orthogonal transformation.

The steps in PCA feature reduction are as follows:

(1) The mean value of the dataset *D* is computed.
(2) A new matrix *S* is formed by subtracting the mean value from *D*.
(3) From *S*, covariance is estimated at *C* = AAT. For data 1 to *l*,
  (20) $$\begin{array}{c} S_{k}\in R^{N},\sum_{k=1}^{l}S_{k}=0\text{ covariance c}=\frac{1}{l}\sum_{i=1}^{l}S_{i}S_{i}^{T}. \end{array}$$
(4) Eigenvalues derived from the covariance matrix are [*V*_1_, *V*_2_,…, *V*_*n*_].
(5) Eigenvectors are found from the covariance matrix.
(6) Vector $D-\bar{D}$, a linear function of eigenvectors, is given by
  (21) $$\begin{array}{c} D-\bar{D}=b_{1}u_{1}+b_{2}u_{2}+\cdots b_{n}u_{n}. \end{array}$$
The symmetric covariance matrix is [*V*_1_, *V*_2_,…, *V*_*n*_].
(7) Large eigenvalues reduce the dataset to $D-\bar{D}=\sum_{i=0}^{l}b_{i}u_{i},\;1<N$.

For a normally distributed dataset, the principal components are independent. The PCA depends on the individual variable screening. The PCA reduces the dimensionality of the dataset and increases interpretability with a reduction in loss of information. The variances are maximized one after the other by developing variables, which are uncorrelated.

### 2.6. Classification

The extracted features were given to machine learning classification algorithms, namely, the Naive Bayes algorithm and dynamic learning vector quantization (DLVQ), for a more accurate classification of the carotid artery ultrasound images.

#### 2.6.1. Naive Bayes Algorithm

Bayes theorem considers an event probability based on prior knowledge of the related conditions with statistical interference. The Naive Bayes classification algorithm is a supervised approach, which depends on the Bayes theorem and assumes that the occurrence of a feature is not dependent on other features in the image. Frequency tables are generated from the dataset, followed by probability-based likelihood table generation and posterior probability calculation based on the Bayes theorem that are performed. Since the dataset followed a normal distribution, Gaussian-based Naive Bayes classification is performed for the dataset images. Weighting the features and structure extension is used to overcome the drawback of the complete feature independence assumption. A kernel function is used to estimate the data distribution instead of following a normal distribution.

For the two-class problem *C*_1_, *C*_2_, samples are indicated by *n* dimensional vectors.

$\overset{⟶}{Y}=\left\{Y_{1},\;Y_{2},\;\ldots Y_{m}\right\}$ containing *m* attributes *A*_1_ to *A*_*m*_ projecting *m* measured values. $\overset{⟶}{Y}$ needs to be found with the highest a posteriori probability. *Y* lies inside the class *C* based on the following condition.

(22) $$\begin{array}{c} P\left(C_{i}|\overset{⟶}{Y}\right)=\;\frac{P\left(\overset{⟶}{Y}|C_{i}\right)P\left(C_{i}\right)}{P\left(\overset{⟶}{Y}\right)}. \end{array}$$

The numerator value should be maximized to get a higher probability, since the probability of getting the *Y* vector is a constant. The assumption that the classes are conditional independent is performed [31, 32].

(23) $$\begin{array}{c} P\left(\overset{⟶}{Y}|C_{i}\right)\;\approx \;\overset{m}{\underset{s=1}{\prod }}P\left(Y_{s}|C_{i}\right). \end{array}$$

The *Y*_1_ and *Y*_2_ probabilities can be obtained from the training set.

Since Naive Bayes assumes that all the dimensions are independent, the classification accuracy is minimum compared to the LVG algorithm.

#### 2.6.2. Dynamic Learning Vector Quantization (DLVQ)

The LVQ is a winner take all Hebbian learning-based supervised learning approach. The algorithm classifies the sample with the same label as the codebook prototype, similar to the nearest neighbor classification. In the training phase, LVQ iteratively goes through the training sample *y*_*j*_, shifted to the nearest available prototype *n*_*m*(*j*)_ nearer or farther from *y*_*j*_. This depends on if the *n*_*m*(*j*)_ is in the same class as *y*_*j*_.

(24) $$\begin{array}{c} n_{m\left(j\right)}=\left\{\begin{array}{c} n_{m\left(j\right)}+\rho \left(y_{j}-n_{m\left(j\right)}\right),\text{if }n_{m\left(j\right)}\text{has label }x_{j},\\ n_{m\left(j\right)}-\rho \left(y_{j}-n_{m\left(j\right)}\right),\text{otherwise}, \end{array}\right. \end{array}$$

*ρ* is between 0 and 1, and it is a step size, which is monotonically reducing [33, 34]. An adaptive version of LVQ is DLVQ, which can acclimate the neuron count to the dataset. It is a computationally less cost approach that has two layers of the fully connected network. The output parallels to one of the classes and matches to a reference vector with weights assigned. The output value is the consolidation of the outcomes of all the output neurons, which signifies the distance between the input sample and the reference vector. The distance helps in weight updating during the training phase and assigning the neuron to the corresponding output during the classification [33]. The frequency-sensitive competitive learning approach is used for training the network. DLVQ is used with global average pooling so that the model parameter count is minimized, thus reducing overfitting.

## 3. Results and Discussions

For the carotid artery ultrasound images, feature extraction, selection, and classification are performed. The results are analyzed with the classification performance measures.

### 3.1. Feature Extraction and Selection

The features extracted from the carotid artery ultrasound images are normalized and plotted for normal images without abnormalities and abnormal images with plaque deposit. From the bar plot, if a particular feature records significant variation for normal and abnormal images, it is considered as a significant feature. If a feature does not show variations for normal and abnormal images, it is considered not a significant feature and is omitted.

**Figure 4:**
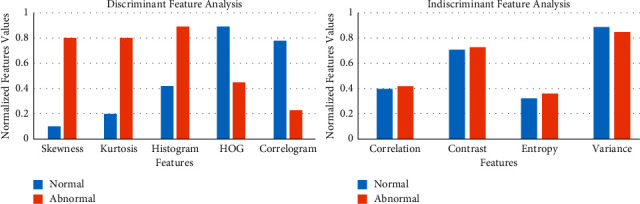
Feature analysis.

Figure 4 shows sample discriminant features such as skewness, kurtosis, histogram, HOG, and correlogram. Features with more variance help in machine learning classification since the values are far apart for normal and abnormal cases. For sample features such as correlation, contrast, entropy, and variance, the normalized feature values are indiscriminate and are hence not useful for machine learning classification.  Table 2 gives the selected features based on discriminant and PCA feature reduction technique for the carotid artery ultrasound images.

The statistical PCA model identifies the discrete pattern in the data and thus selects only the required features. The input neurons of the classification algorithms are powered by the potential features.

### 3.2. Classification Performance Measures

The selection of appropriate performance measures for evaluating the machine learning classification algorithms provides faith for its real-time application. Since the data are imbalanced, applying more measures provides a clear insight into the algorithm usage. Classification of the carotid artery ultrasound images is a binary class problem, correct classification as fitting to the class being true positive (TP), correct classification as not fit in the class as true negative (TN), wrongly categorized to a class false positive (FP), and misclassified to a class as false negative (FN). The general efficiency of the model is given by

(25) $$\begin{array}{c} \text{accuracy}=\frac{\text{TP}+\text{TN}}{\text{TP}+\text{TN}+\text{FP}+\text{FN}}\text{ .} \end{array}$$

Agreement of the labels with positives is given by

(26) $$\begin{array}{c} \text{precision}=\frac{\text{TP}}{\text{TP}+\text{FP}}. \end{array}$$

Sensitivity and recall give the efficiency of identifying positive labels. The data out of the classifier and the positive labeled ones' relationships are measured by F score. Negative label identification performance is measured by specificity. Recall is given by TPR, and probability is given by FPR. Area under the ROC curve (AUC) gives false classification identification presentation of the model, where 1 depicts a *n* ideal model [35, 36].

(27) $$\begin{array}{c} \text{recall}=\frac{\text{TP}}{\text{TP}+\text{FN}},\\ \text{precision}=\frac{\text{TP}}{\text{TP}+\text{FP}},\\ \text{f score}=2\times \frac{\text{precision}\times \text{recall}}{\text{precision}+\text{recall}},\\ \text{specificity}=\frac{\text{TN}}{\text{TN}+\text{FP}},\\ \text{AUC}=\left(\frac{1}{2}\right)\left(\frac{\text{TP}}{\text{TP}+\text{FN}}+\frac{\text{TN}}{\text{TN}+\text{FP}}\right),\\ \text{Accuracy}=\frac{\text{TP}+\text{TN}}{\text{TP}+\text{TN}+\text{FP}+\text{FN}}. \end{array}$$

These parameters denote the classification model performance. The classification models used ReLU activation function. ReLU is a nonlinear activation function, which does not activate all neurons at the same time. It is fast and easy and converges faster compared to other activation functions.

### 3.3. Machine Learning

The database comprised of 202 normal images without any plaque deposit and 159 images with plaque deposit. The selected features are provided as the input neurons of the ML approaches. Box-Cox plot transforms the data so that it can be similar to a normal distribution. Considering the presence of normally distributed errors, a hypothesis test can be performed. The gain curve gives the performance of the machine learning models with randomly taken data. The curve analyzes the percentage of the target compared with a group with the highest probability. The receiver operating characteristic (ROC) curve provides the sensitivity vs. specificity relation for the probable cutoff values in all classification thresholds. The ordinal curve gives the contribution of the features and their significance. It helps in the data to rely on one final side.

**Figure 5:**
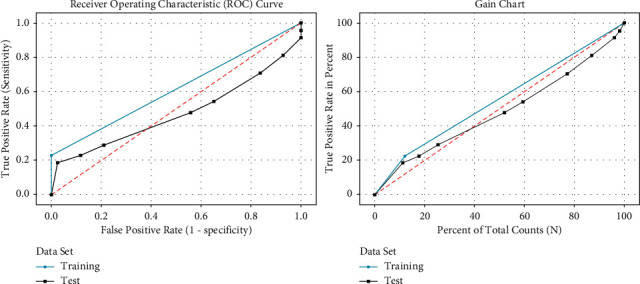
ROC curve and gain chart of Naive Bayes algorithm.

Figure 5 gives the ROC curve and gain chart of the Naive Bayes classification. It has recorded an area under the curve training of 61.46% and 47.50% for testing. The gain chart gives the whole positive rate percentage to the total count percentage.

**Figure 6:**
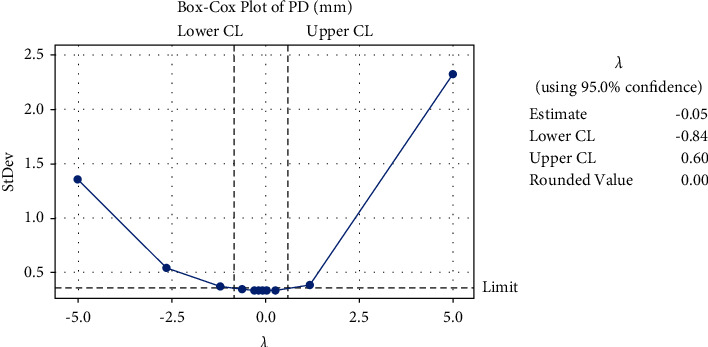
Box-Cox plot of plaque diameter.

**Figure 7:**
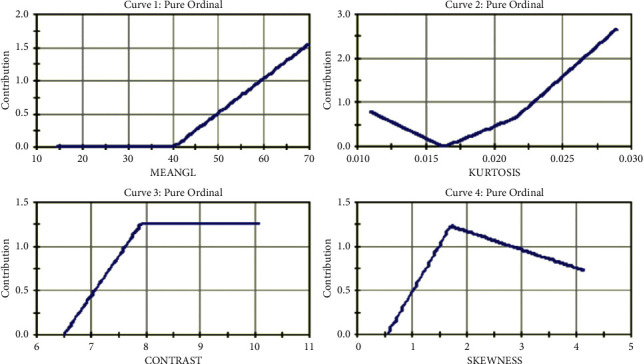
Pure ordinal curve.

Figure 6 gives the Box-Cox plot for the plaque diameter, which is a linearity plot giving the correlation between the transformed StDev and the given lambda values.  Figure 7 gives the pure ordinal curve for sample features meangl (mean gray level), kurtosis, contrast, and skewness.

Table 3 provides the confusion matrix of the applied ML techniques, namely, Naive Bayes and DLVQ, performed in 361 carotid artery ultrasound images.

Table 4 gives the comparison of the Naive Bayes and dynamic learning vector quantization approaches using the classification performance measures.

Table 5 separately gives the accuracy of the Naive Bayes and DLVQ algorithms with the extracted sixty-three features and the selected twenty-two features. Improved accuracy is found in both the machine learning algorithms on using only the selected features.

Classification of hard and soft plaques will aid in diagnosing dangerous plaque types and may be implemented as a future work. A patient-specific model with more measurements from a single patient including boundary conditions, pressure, geometry, 3D images, and thermal images may be implemented to improve diagnostic performance.

## 4. Conclusions

Sixty-three features consisting of texture, shape, histogram, correlogram, and morphology were extracted from the carotid artery ultrasound images. Principal component analysis (PCA) and feature analysis by feature significance-based feature reduction were performed to minimize overfit and underfit problems. The robust subset from the extracted features reduces model complexity and computation and improves the efficiency of the classification task. Classification of Naive Bayes and DLVQ algorithms is performed using the extracted and selected features. DLVQ with the selected features recorded an accuracy of 91.68%, specificity 98.51%, sensitivity 83.01%, precision 97.77, F score 89.78%, and AUC 98.14%. DLVQ has proven to give improved performance compared to the DLVQ classification approach, on using the selected extracted features.

## Figures and Tables

**Table 1:** List of extracted features.

Sl. no	Feature type	Features
1	Texture features (33)	Gray-level co-occurrence matrix (GLCM)—inertia, energy, correlation, contrast, entropy, and homogeneity
		Gabor wavelet
		Statistical features—mean, median, kurtosis, and skewness
		Local binary pattern (LBP)—textures spatial structure
		Gray-level difference statistics (GLDS)—mean, entropy, contrast, angular second moment, and homogeneity
		Fractal dimension texture analysis (FDTA)
		Radial and angular sum of discrete Fourier transform for Fourier power spectrum
		Neighborhood gray-tone difference matrix (NGTDM), given by strength, complexity, coarseness, and contrast
		Absolute gradient—mean, variance

2	Shape features (5)	Sharpness
		Complexity
		Length irregularity
		Aspect ratio and circularity

3	Histogram and correlogram features (10)	Gray-level histogram of segmented ROI of the carotid image—for 32 same measurements, bins were computed
		Plaque histogram represents plaque characterization
		Multiregion histogram—to check whether plaque outer region signifies disease progression
		Grayscale median (GSM) derived from the histograms first-order statistics, and entropy represents echogenicity
		Histogram of oriented gradient (HOG)—gradient magnitude and orientation
		Correlogram—statistics and spatial distribution of the features
		Texture and shape features—normalized; histogram and correlogram features—used without normalization

4	Morphology features (15)	Mean probability density functions (PDFs), mean cumulative distribution functions (CDFs)
		Plaque power spectra for all the three intensity variations (low, medium, and high)
		Shape, connectivity, and convexity
		Plaque size, lipid core, and presence of intraplaque hemorrhage
		Smooth lumen surface—no risk; rough lumen—leads to stroke
		Plaque volume

**Table 2:** List of selected significant features.

Sl. no	Selected features	Description
1	Texture	Spatial arrangement of image intensity
2	Spatial structure	Exploit location information
3	Skewness	Extent to which a distribution differs from a normal distribution
4	Kurtosis	Pixel intensity distribution
5	Histogram	Pixel distribution as a function of tonal variation
6	Correlogram	Spatial correlation of intensity changes with distance
7	HOG	Count incidences of gradient alignment in localized regions of an image
8	Gabor wavelet	Frequency-wise intensity variation check in specific direction
9	Angular 2nd moment	Textural uniformity in image
10	Shape	Shape characteristics
11	Sharpness	Degree of clarity in both coarse and fine image detail
12	Length irregularity	Irregularities of the length of structures in an image
13	Mean probability density function	Probability that the region brightness is less than or equal to a specified brightness value
14	Grayscale median	Median of grayscale intensities
15	Multiregion histogram	Checks whether plaque outer region signifies disease progression
16	Arterial wall ROI's randomness	Randomness present in the artery wall
17	Absolute gradient	Directional change in intensity
18	Angular and radial sum of discrete Fourier transform for Fourier power spectrum	Fourier power spectrum's Fourier transform
19	Coarseness	Type of texture feature
20	Convexity	Convex curves present in an image
21	Connectivity	Connectivity among pixels
22	Plaque volume	Plaque volume measure

**Table 3:** Confusion matrix of the ML algorithms.

	Naive Bayes		DLVQ	
	Actual positive (1)	Actual negative (0)	Actual positive (1)	Actual negative (0)
Predicted positive (1)	121	24	132	3
Predicted negative (0)	38	178	27	199

**Table 4:** Performance comparison of classification of carotid artery ultrasound images using ML approaches.

Algorithm	Accuracy	Specificity	Sensitivity	Precision	F score	AUC
Naive Bayes	82.82	88.11	76.1	83.44	79.60	85.77
DLVQ	91.68	98.51	83.01	97.77	89.78	98.14

DLVQ has recorded improved performance in terms of AUC, F score, precision, sensitivity, specificity, and accuracy compared with the Naive Bayes algorithm.

**Table 5:** Comparison of DLVQ performance with all extracted and selected features.

Accuracy	With all extracted features	With selected features
Naive Bayes	80.91	82.82
DLVQ	88.72	91.68

## Data Availability

The data are not from any public database. So the authors will not be able to share the data.
